# Effects of Tetrahydrolipstatin on Glioblastoma in Mice: MRI-Based Morphologic and Texture Analysis Correlated with Histopathology and Immunochemistry Findings—A Pilot Study

**DOI:** 10.3390/cancers16081591

**Published:** 2024-04-21

**Authors:** Sabine Wagner, Christian Ewald, Diana Freitag, Karl-Heinz Herrmann, Arend Koch, Johannes Bauer, Thomas J. Vogl, André Kemmling, Hubert Gufler

**Affiliations:** 1 Department of Neuroradiology, Marburg University Hospital, Philipps University, 35043 Marburg, Germany; andre.kemmling@uk-gm.de; 2 Department of Neuroradiology, Institute for Diagnostic and Interventional Radiology, Jena University Hospital, Friedrich Schiller University, 07747 Jena, Germany; 3 Department of Neurosurgery, Brandenburg Medical School, Campus Brandenburg, 14770 Brandenburg a. d. Havel, Germanyj.bauer@uk-brandenburg.de (J.B.); 4 Department of Neurosurgery, Section of Experimental Neurooncology, Jena University Hospital, Friedrich Schiller University, 07747 Jena, Germany; diana.freitag@med.uni-jena.de; 5 Medical Physics Group, Institute for Diagnostic and Interventional Radiology, Jena University Hospital, Friedrich Schiller University, 07743 Jena, Germany; karl-heinz.herrmann@med.uni-jena.de; 6 Department of Neuropathology, Charité—Universitätsmedizin Berlin, Corporate Member of Freie Universität Berlin, Humboldt-Universität zu Berlin, and Berlin Institute of Health, Charité University Medicine, 10117 Berlin, Germany; 7 Department of Diagnostic and Interventional Radiology, Goethe University Hospital Frankfurt, 60590 Frankfurt am Main, Germany; t.vogl@em.uni-frankfurt.de (T.J.V.); hgufler@gmx.de (H.G.)

**Keywords:** tetrahydrolipstatin, orlistat, magnetic resonance imaging, glioblastoma, texture analysis, animal experiment

## Abstract

**Simple Summary:**

Glioblastomas are the most aggressive brain tumors, and affected patients still only have an extremely poor prognosis with today’s therapeutic options. Further developments are still necessary, particularly in therapeutic approaches. Orlistat can act as an antitumor agent as it inhibits fatty acid synthase, decreases tumor cell proliferation, and stimulates tumor cell apoptosis. Investigations conducted on breast, pancreatic, hepatic, and colorectal tumors showed that FASN—fatty acid synthase, a protein that catalyzes the de novo synthesis of long-chain fatty acids—is strongly upregulated. Using an animal model, we tested whether the drug’s effects could be demonstrated visually, quantitatively, and by texture analysis based on MR studies. Histology and immunochemistry were used as references. The key results of the present study are as follows: Firstly, a significant difference was found between orlistat-treated and untreated tumors in MRI studies based on morphology and texture analyses. Secondly, the expression of FASN was reduced in the orlistat group, which, however, did not result in a higher apoptosis rate in the treatment group. Our findings suggest that some effects of orlistat on tumor cell proliferation must have taken place during therapy. Further studies should investigate the effects of FASN inhibition when combined with targeted therapies.

**Abstract:**

Background: This study aimed to investigate the effects of tetrahydrolipstatin (orlistat) on heterotopic glioblastoma in mice by applying MRI and correlating the results with histopathology and immunochemistry. Methods: Human glioblastoma cells were injected subcutaneously into the groins of immunodeficient mice. After tumor growth of >150 mm^3^, the animals were assigned into a treatment group (n = 6), which received daily intraperitoneal injections of orlistat, and a control group (n = 7). MRI was performed at the time of randomization and before euthanizing the animals. Tumor volumes were calculated, and signal intensities were analyzed. The internal tumor structure was evaluated visually and with texture analysis. Western blotting and protein expression analysis were performed. Results: At histology, all tumors showed high mitotic and proliferative activity (Ki67 ≥ 10%). Reduced fatty acid synthetase expression was measured in the orlistat group (*p* < 0.05). Based on the results of morphologic MRI-based analysis, tumor growth remained concentric in the control group and changed to eccentric in the treatment group (*p* < 0.05). The largest area under the receiver operating curve of the predictors derived from the texture analysis of T2w images was for wavelet transform parameters WavEnHL_s3 and WavEnLH_s4 at 0.96 and 1.00, respectively. Conclusions: Orlistat showed effects on heterotopically implanted glioblastoma multiforme in MRI studies of mice based on morphologic and texture analysis.

## 1. Introduction

Glioblastoma multiforme (GBM) is one of the most aggressive primary brain tumors, with a median survival time of 10–18 months despite maximal therapy and less than 5% of patients alive at 5 years [1]. Nevertheless, some patients with GBM survive longer than 36 months [2,3]. Reasons for its poor prognosis include the infiltrative nature of GBM, which precludes disease cure even after supramaximal resection, and its inherent resistance to radiation and chemotherapy [4]. This resistance seems to be linked to intricate alterations in biochemical and signaling pathways driven by genetic determination within the heterogenous tumoral cells and their microenvironments [5,6].

In most animal models, human glioblastoma cells are either implanted orthotopically into the brain or, exceptionally, heterotopically into the subcutaneous tissue of immunocompromised mice. How to quantify tumor growth and how to assess treatment response non-invasively remains a major challenge for all tumor models, because disease progression cannot be reliably characterized by an increase in lesion size alone [7]. Additional criteria related to changes in cell density, heterogeneity, vascularization, and tumor necrosis formation are important as well [8,9]. Such tissue characteristics may be amenable to MR image texture analysis, a technique that has gained increased attention in recent years and is used as an additional, quantitative tool to characterize tumors and evaluate tumor response to therapy [10,11,12,13].

The fatty acid synthase (FASN) inhibitor tetrahydrolipstatin (orlistat), used to treat obesity, represents a potential new option for antitumor therapy [14]. The overexpression of FASN has been described in many tumor types, including glioblastomas [15]. By inhibiting FASN and thus disrupting the lipid metabolism pathway, cell growth is reduced through the activation of proapoptotic signaling cascades, ultimately leading to cell death.

In this study, we tested the potential antiproliferative and proapoptotic effect of orlistat in heterotopically transplanted glioblastoma cells in mice. The therapy group was compared to controls intraperitoneally injected only with saline. The interior structure of all tumors was assessed visually, quantitatively, and by texture analysis based on MR images. Histology and immunochemistry were used as references.

## 2. Materials and Methods

This study was performed in accordance with the European Convention for Animal Care and Use of Laboratory Animals and was approved by the local authorities (TVL 02-029/13).

### 2.1. Cell Culture and Xenograft Transplantation

A172 human glioblastoma cells were cultured in DMEM (Dulbecco’s modified Eagle medium, GIBCO^®^DMEM, Darmstadt, Germany) and supplemented with 10% fetal bovine serum. Cells were routinely passed with trypsin (0.05%)/EDTA. For xenotransplantation, the cells were separated with trypsin (0.05%)/EDTA for up to 5 min, washed with 1× DPBS (Dulbecco’s phosphate-buffered saline, GIBCO^®^ DPBS, Darmstadt, Germany), and adjusted to 1 × 10^7^ cells/mL.

The immunodeficient mice (Harlan Laboratories, Rossdorf, Germany) were maintained under specific pathogen-free conditions. The xenografts were established in 8- to 10-week-old female animals by the subcutaneous inoculation of 1 × 10^6^ A172 cells into both flanks. A single dose consisted of 100 µL cell suspension mixed with 100 µL growth factor-reduced Matrigel (BD Matrigel™ Basement Membrane Matrix, BD Biosciences, Erembodegem, Belgium).

### 2.2. In Vivo Treatment with the FASN Inhibitor Orlistat

**Figure 1:**
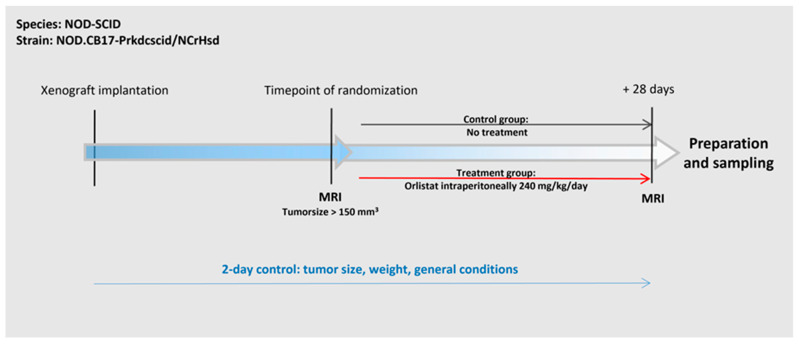
Schematic timeline of the experiment.

When the xenograft reached a size of ≥150 mm^3^, the animals were randomly assigned into two groups. Group 1 (treatment group) received daily intraperitoneal injections of 240 mg/kg body weight orlistat (Ratiopharm, Ulm, Germany) dissolved in a 30 µL vehicle consisting of 33% ethanol and 66% polyethyleneglycol/0.9% sodium chloride (PEG400) over a period of 28 days. Group 2 (control group) consisted of mice that received intraperitoneal saline injections only. MRI examinations were performed at the time of randomization, and before the animals were euthanized. Finally, the tumors were sampled and processed for RNA, protein, and histology/immunohistochemistry studies (Figure 1).

### 2.3. Animal Preparation for MRI Examination and MRI Protocol

Temperature, cardiac frequency, and respiration were monitored. For anesthesia, a gas mixture of 2% isoflurane and oxygen was used. A dedicated volume coil for small animal imaging with a linearly polarized Litz volume resonator with a 38 mm inner diameter and 33 mm homogeneous RF window length (Doty Scientific Inc., Columbia, SC, USA) was used.

Briefly, 3D T1w MR volumes and a T2w, single-slab 3D TSE sequence were obtained. Then, Gd-DTPA (0.1 mmol/kg) was manually injected into a tail vein, and a second 3D T1w MR volume set was acquired immediately thereafter. The parameters of the sequences are presented in Table 1.

### 2.4. MR Image Evaluation and Texture Analysis

Qualitative and quantitative image analyses were based on reconstructions of the 3D volume datasets with 0.3 mm thick slices using the software tool XrayLine™ Workstation 2.0. The image with the largest cross-sectional lesion diameter was chosen for analysis.

The visual qualitative assessment of each MR measurement was adapted from the VASARI (Visually AcceSAble Rembrandt Images) MRI feature set [16] and, assuming that the entire tumor may comprise a contrast-enhancing component (the proportion of the entire tumor that is contrast-enhancing), a non-contrast-enhancing component, and a necrotic component (necrosis is defined as a region within the tumor that does not enhance or show markedly diminished contrast enhancement, is high on T2w and low on T1w images, and has an irregular border), included the following features: (a) the intensity of contrast enhancement (n/a, none, mild, or marked); (b) the contrast enhancing portion of the tumor (n/a, none, <1/3, 1/3–2/3, >2/3, or all); (c) the portion of necrosis (none, <1/3, 1/3–2/3, >2/3, or all); (d) the signal intensity (SI) of the margin on T1w precontrast images compared to the neighboring muscle; (e) the thickness of the contrast enhancing margin (none, thin, or thick); (f) the pattern of tumor growth (concentric with increasing radius over time around an assumed common center vs. eccentric tumor growth with deviation from the assumed center point); and (g) the presence of cysts, hemorrhage, and feeding vessels.

For the quantitative analysis of the SI, a polygonal region of interest (ROI) was drawn, covering the entire lesion on T2w and T1w pre- and postcontrast images. The mean value was normalized by calculating the quotient of the mean value of the lesion and the mean value of the neighboring muscle. The volume of the lesion was calculated according to Cavalieri’s method (total volume [mm^3^] = sum of all cut surface areas [mm^2^] × slice thickness [mm]). For tumor texture analysis, wavelet transform energy parameters, obtained with the freely available MaZda software (Version 4.6, Institute of Electronics, Technical University Lodz, Poland), were used [17].

### 2.5. Pathological Analysis

All mice were sacrificed after the last MRI examination. The tumors were fixed in 10% phosphate-buffered formalin, embedded in paraffin, serially sectioned at 3 µm, and stained with hematoxylin–eosin (HE) for histological examination. In addition, we immunohistochemically stained the sections for glial fibrillary acidic protein (GFAP), microtubule-associated protein 2 (MAP2), p53, and Ki67. For each case, the Ki67 proliferation activity was determined, and a fixed value was set. The value results from the percentage of proliferating cells per one high-power field (HPF) (1 HPF = 0.26 mm^2^). Benign tumors usually show a Ki67 proliferation activity of 1 to 2%, while high-grade CNS tumors (grade 3 or 4) show proliferation activities of 10% or more. A neuropathologist carried out the histologic evaluation.

### 2.6. Western Blot Analysis

Western blot analysis was performed to examine the effect of orlistat on tumor progression, apoptotic effects, and blockage of the FASN by measuring the apoptosis-regulating proteins caspase-3, caspase-8, polyadenosine diphosphate ribose polymerase-1 (PARP-1), and apoptotic protease activating factor-1 (APAF1), as well as FASN, nuclear factor of kappa light polypeptide gene enhancer in B-cell inhibitor alpha (IκB-alpha), and nuclear factor kappa-light-chain enhancer of activated B cells (NF-κB) (Table 2 and Table 3). Glyceraldehyde-3-phosphate dehydrogenase (GAPDH) was used for normalization.

For Western blot analysis, the proteins were extracted from the tumor tissues of the mouse flanks. The tissues were homogenized in 500 µL RIPA buffer and vortexed every 10 min during a 30 min incubation on ice. After centrifugation at 12,000 rpm at 4 °C for 10 min, the supernatants were transferred to a new tube and stored at −80 °C until further use.

For Western blotting, 20 µg proteins, as determined by the bicinchoninic acid (BCA) protein assay kit (Thermo Fisher Scientific Inc., Waltham, MA, USA), were separated by sodium dodecyl sulfate (SDS)–polyacrylamide gel electrophoresis on 10% polyacrylamide gels. Proteins were electrotransferred onto Immobilon-P polyvinylidene fluoride PVDF membranes (Merck Millipore, Merck KGaA, Darmstadt, Germany), blocked for 2 h at room temperature with 5% fetal bovine serum (BSA) or 5% dry milk in TRIS-buffered saline, 0.05% Tween-20 (TBS-T, Sigma, Roedermark, Germany), depending on the secondary antibodies used, and probed for 1 h with primary antibodies. The membranes were washed three times for 10 min in TBS-T and incubated for 1 h at room temperature with secondary antibodies. After washing the membranes in TBS-T, the proteins were visualized using Immobilon Western HRP Substrate (Merck Millipore), according to the manufacturer’s instructions. Signal strengths were recorded using a chemiluminescence scanner (LAS3000, Fujifilm Medical Systems USA, Inc., Stamford, CT, USA).

### 2.7. Statistical Analysis

Statistical analysis was performed using the open source statistics package R (R Foundation for Statistical Computing, Vienna, Austria) with RStudio 4.3.6, employing dedicated packages. Bar plots were used for data presentation, applying the *ggplot2* and *ggpubr* packages in RStudio. For continuous data, the Wilcoxon test was applied, and categorical variables were derived from the qualitative analysis, the chi-squared test, or Fisher’s exact test, as appropriate. The significance level was set at 0.05. The mixed ANOVA method was used to test if the means of the features obtained by texture analysis between each other or the means between the treatment and the control group differed, and if there were any interaction effects between groups and features. The relative importance of the wavelet energy transform features provided by the MaZda software (Version 4.6) was determined using a logistic regression model and receiver operating curve (ROC) analysis (with the pROC package).

## 3. Results

A total of thirteen tumors in nine animals were included in this study and randomized in the treatment group (five mice, unilateral–bilateral tumor growth 4:1) and the control group (four mice, unilateral–bilateral tumor growth 1:3). MRI examination was performed twice, the first at the time of randomization and the second 19–34 days (mean 28 days) later; immediately afterward, the animals were euthanized for histological workup.

### 3.1. MR Image Evaluation and Texture Analysis

**Figure 2:**
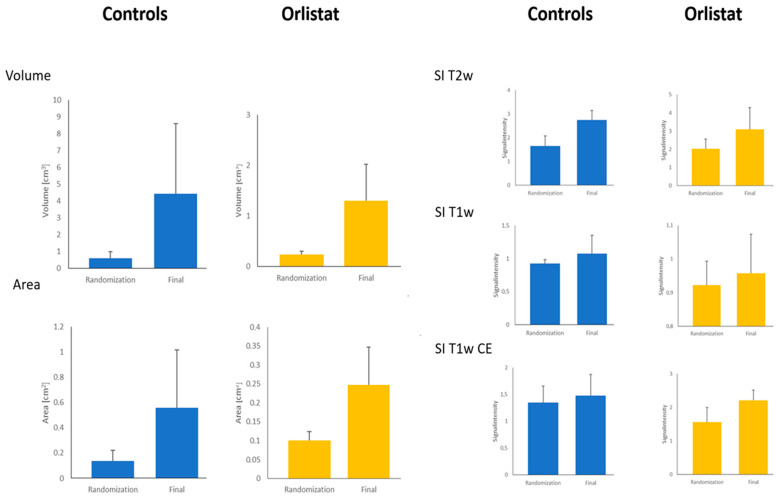
Overview of the results of quantitative MR analysis. MRI was performed twice, the first at the time of randomization and the second immediately before the animals were euthanized for histological and immunochemical workup (final). Abbreviations: SI = signal intensity; CE = contrast enhancement.

The volumes of the lesions did not differ between the treatment and the control groups (Figure 2), neither at the time of randomization nor at the final MRI examination. The SI of T1w, T2 w, and contrast-enhanced T1w images only showed a trend toward higher values for the treatment group (*p* > 0.5, *p* > 0.3, and *p* > 0.4, respectively).

**Figure 3:**
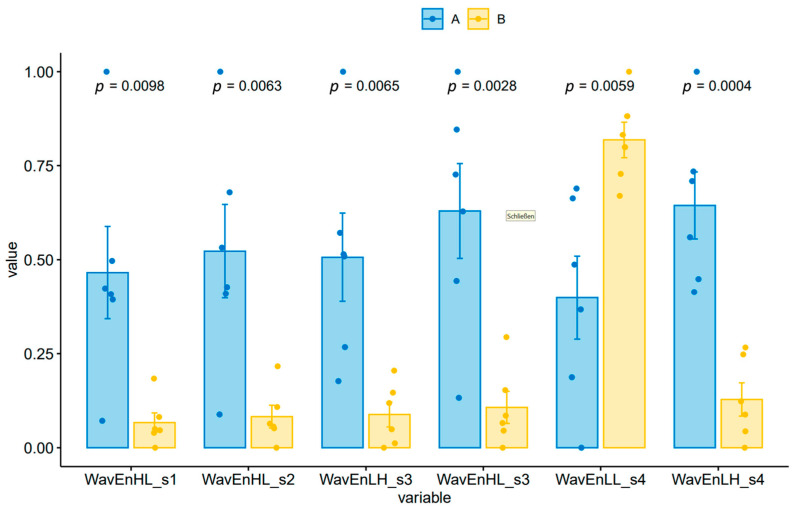
Results of wavelet energy transform analysis of T2w images showing significant differences between the groups for all variables. Wilcoxon test was used for statistical analysis. A = control group (blue bars); B = treatment group (yellow bars); *x*-axis: Wav stands for “wavelet transform”, EN for “energy”, L and H combinations for high-pass (H) and low-pass (L) filters, and s for stage (level) of transform. Note: values on the *y*-axis are normalized into a range between 0 and 1 for better comparability.

**Figure 4:**
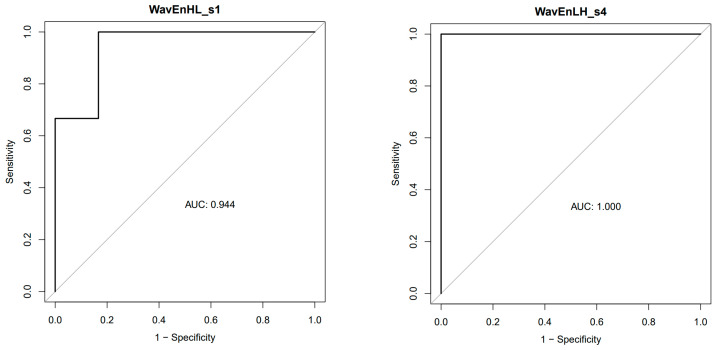
ROC analysis showing the feature with the highest (WavEnLH_s4) and the lowest (WavEnHL_s1) area under the curve (AUC). WavEnHL s1 = wavelet energy; H = high-pass filter; L = low-pass filter; s = stage (level) of transform.

However, the wavelet energy texture analysis of the T2w images showed significant differences between the two groups (Figure 3). With the MaZda software, feature weighting and feature extraction were performed automatically, offering (in our case six) features with the highest Fisher coefficient. Applying the mixed ANOVA method, we found interaction effects (*p* < 0.0001) between the factor “treatment and control group” and the features from texture analysis. The highest difference between the treatment and controls was found for the feature “WavEnLH_s4”. The relative importance of each of the six selected features was calculated by ROC analysis. The area under the curve after ROC analysis was highest for the features WavEnLH_s4 (1.00), followed by WavEnHL_s5 and WavEnHL (AUC 0.97 and 0.94) (Figure 4).

Feature extraction for T1w before and T1w images after administering the contrast agent yielded low Fisher coefficients, and according to MANOVA, the difference between the treatment and the control group did not reach statistical significance.

**Figure 5:**
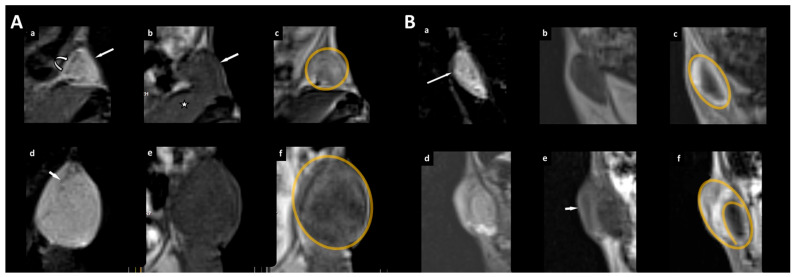
Example of unhindered tumor growth of the xenograft in the right flank of an immunodeficient female mouse (**A**) and example of a xenograft treated with orlistat (**B**). (**A**,**B**) (**a**–**c**) timepoint of randomization, (**d**–**f**) last MR examination before sacrificing the animal for histological workup. A triplet of the magnified tumor is given with a coronal reconstruction from the 3D T2w volume dataset with fat saturation (**a**,**d**) and of the T1w dataset (before contrast administration (**b**,**e**); after contrast administration (**c**,**f**) with a 0.3 mm slice thickness. (**A**) The straight arrow (**a**) indicates the xenograft. The margin (curved arrow) of the xenograft is visible with high signal intensity on T2w. The xenograft is isointense to the neighboring muscle (asterisk) on T1w images (**b**) with marked contrast enhancement (**c**). After unhindered tumor growth, the area on the image with the largest cross-sectional diameter of the lesion increased from 0.18 cm^2^ (**a**–**c**) to 0.60 cm^2^ (**d**–**f**). The pattern of tumor growth is concentric (the tumor border is marked in orange in **c**,**f**). In the T2w image (**d**), areas with linear signal voids within the tumor (arrow) are seen as a correlate for histologically confirmed neovascular proliferation. (**B**) Tumor (arrow in **a**) before (upper row) and after treatment with orlistat (lower row). In contrast to unhindered tumor growth in (**A**) with a concentric growth pattern, the tumor shows eccentric growth with a focus on the lateral border and upper outer quadrant after treatment with orlistat (arrow in **e**; tumor border is marked in orange in **c**,**f**).

Evaluating the qualitative MR parameters, we found that areas of necrosis were only in the control group (3/7), whereas neovascular proliferation was observed in both groups (1/6 in the treatment group vs. 4/7 in the control group) (Table 4). Fisher’s exact test revealed that the pattern of tumor growth (eccentric versus concentric) was significantly different between the two groups (*p* < 0.05) (Figure 5; Table 4).

### 3.2. Histological and Laboratory Examination

**Figure 6:**
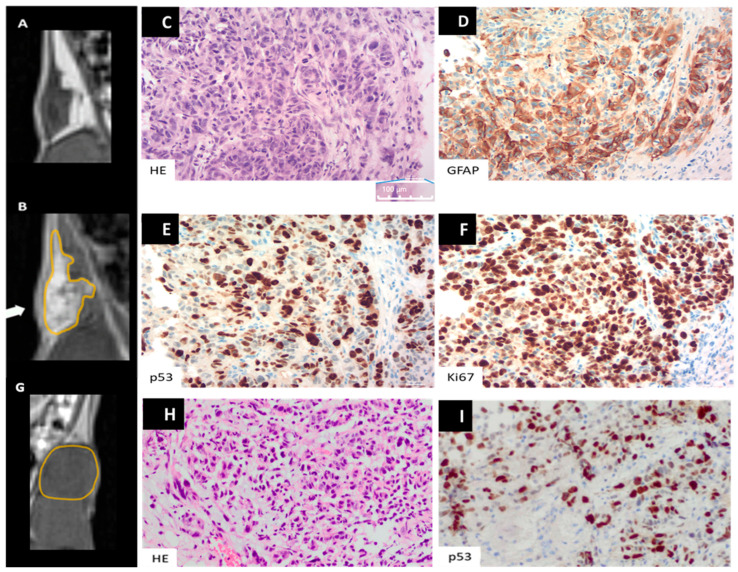
Example of histological workup of a xenograft treated with orlistat (**C**–**F**) and one with unhindered tumor growth (**H**,**I**). (**A**,**B**) Magnified MR image of coronal reconstructions from the 3D T1w volume dataset before (**A**) and after contrast administration (**B**). The tumor in the left groin (arrow) has an area of 0.15 mm^2^. Note the eccentric tumor growth after treatment with orlistat with a focus on the medial border in the lower quadrant (arrow in **B**; tumor border is marked in orange in **B**). (**C**–**F**,**H**,**I**) Histological examination revealed a high density of pleomorphic cells interspersed with endothelial cell-proliferating neovascularization. At higher magnification (**C**,**H**), neoplastic cells stained with H&E showed nuclear polymorphism and frequent mitotic figures in both groups (black arrows in **C**). Immunohistochemistry revealed glial fibrillary acidic protein (GFAP) (**D**) and nuclear accumulation of p53 in both groups (**E**,**I**). High Ki-67 expression is shown in (**F**), with cancer nuclei being stained (brown). There is tumor cell positivity in 90% of the cells: Ki-67 labeling index = 90%. Note: The size reference bar is shown in the lower right corner of the histological images and corresponds to 100 µm (magnified in **C**). (**G**) Magnified MR image of coronal reconstructions from the 3D T1w volume dataset of an example of unhindered tumor growth of the xenograft. In contrast to (**A**) and (**B**), note the concentric growth pattern when tumor growth is unhindered (the tumor border is marked in orange).

All tumors were solid and densely cell-packed and showed high mitotic and proliferative activity (Ki67 > 10% to 50%). Regarding the morphology, astroglial (glioblastoma-typical) differentiation was evident. Necrosis was only found in the control group (3/7), and neovascular proliferation was observed in both groups (1/6 in the treatment group vs. 4/7 in the control group) (Table 5; Figure 6).

**Figure 7:**
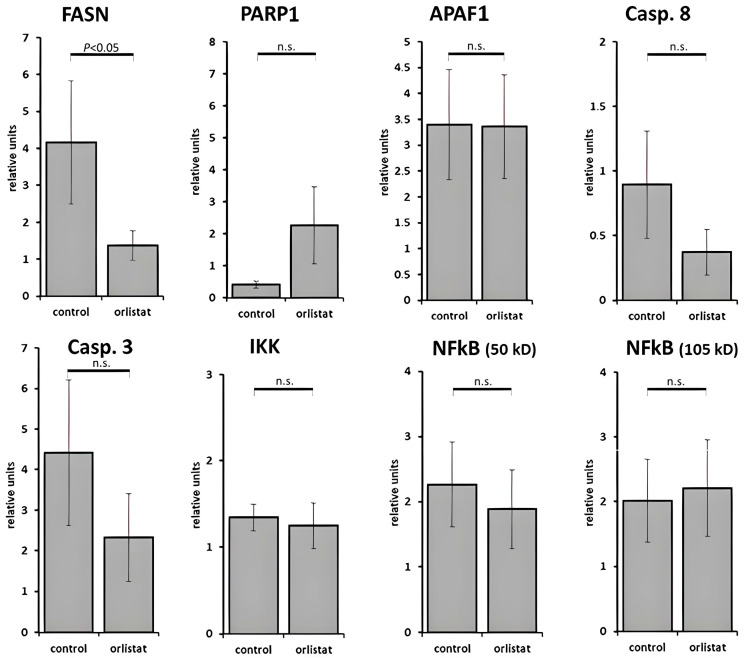
Expression of proapoptotic and proproliferative proteins as well as the fatty acid synthase complex in the tumor samples obtained and analyzed using Western blotting. The box blots show the mean relative light density units, which depend proportionally on protein expression, compared to glyceraldehyde-3-phosphate dehydrogenase (GAPDH) in the two groups. A reduced mean expression of fatty acid synthase (FASN) was found in the orlistat group (*p* < 0.05), whereas the apoptosis rate and the proliferation rate did not differ significantly between the treatment and the control groups. Fisher’s exact test was used. Abbreviations: PARP1 = polyadenosine diphosphate ribose polymerase-1; APAF1 = apoptotic protease activating factor-1; Casp. 3 = apoptosis-regulating proteins caspase-3; Casp. 8 = apoptosis-regulating proteins caspase-8; IKK = IkappaB kinase; NF-κB = nuclear factor kappa-light-chain enhancer of activated B cells; n.s. = non-significant.

Using Western blotting, a reduced mean expression of FASN was found in the orlistat group (*p* < 0.05) (Western blots are provided in the supplement: Supplementary Figure S1), whereas the apoptosis rate and the proliferation rate did not differ significantly between the treatment and the control groups (Figure 7).

## 4. Discussion

The key results of the present study are as follows: Firstly, a significantly different heterogeneity of the internal structure between orlistat-treated and untreated tumors was seen on T2w MR images using texture analysis. Secondly, upon visual MR analysis, the pattern of tumor growth was eccentric in the orlistat group and concentric in the controls. Finally, the expression of FASN was reduced in the orlistat group, which, however, did not result in a higher apoptosis or proliferation rate in the treatment group at the time of tumor harvesting.

Normal human tissues use dietary fat to synthesize new structural lipids, while de novo fatty acid synthesis is maintained at low levels [14]. On the other hand, investigations conducted on malignant tumors showed that the de novo synthesis of long-chain fatty acid FASN is strongly upregulated [18,19,20,21,22,23]. Interestingly, inhibition of de novo lipogenesis with orlistat in tumor models has been shown to reduce tumor regrowth and increase sensitivity to chemotherapy [24,25,26,27,28,29]. Initial results are also available for using orlistat to treat glioblastomas. Guo et al. showed that the increased cell proliferation enhances the need for fatty acids in glioblastoma cells, to provide enough phospholipids for membrane biogenesis in the rapidly proliferating tumor cells [30]. Grube et al. demonstrated that the overexpression of FASN is correlated with the WHO grade of the tumor and orlistat possesses in vitro antitumor qualities in high-grade glioma cells [15]. By inhibiting FASN, palmitate is depleted, which alters the synthesis of membrane phospholipids. Orlistat induced apoptosis within 24 h of incubation in the cell culture and specifically suppressed cell growth and angiogenesis.

In our study, there was a clear inhibitory effect on FASN in the orlistat group, which showed the lowest protein expression, in contrast to the untreated control group. However, contrary to the study by Grube et al., in our in vivo study, the proliferative rates and apoptosis rates did not differ significantly between the treatment and control groups. In a comparative glucose tracer study with the FAS inhibitor C75 in MIA PaCa-2 cells, Harris et al. found that C75 and luteolin both increase cholesterol synthesis from glucose-derived acetyl-CoA as the alternate route of acetate utilization when flux through FAS is blocked [31]. We hypothesize that in our experiment, sterol synthesis replaced de novo fatty acid production, and thus the tumor cells may have found an alternative pathway for regrowth and proliferation.

Furthermore, we found that the tumor volumes increased in both of our groups over time but were stronger in the control group, albeit not statistically significant. However, the pattern of tumor growth was significantly different: While tumor growth remained concentric in the control group (Figure 4), an eccentric growth behavior with a local focus was observed in the treatment group (Figure 5). Interestingly, Aliewa et al. found in their experimental study on mice glioma that two different types of border configurations contributed to tumor cell spreading through distinct invasion patterns: an invasive margin that executes slow but directed invasion and a diffuse infiltration margin with fast but less directed movement [32]. Based on these considerations, we assume that if cells continue to grow unhindered, there is concentric growth, with all cells around the edge showing a comparable potential for dividing or proliferating. In the treatment group, there was a temporary, partial arrest of the tumors showing only eccentric growth. We attribute this observation to a possible intratumoral effect of orlistat due to partial tumor suppression, which may have occurred at an early stage during treatment. In addition, structural changes in tumor appearance may well be supported by a replacement of de novo fatty acid synthesis with that of sterol precursors.

This significantly different MR pattern of tumor growth between the two groups was corroborated by the texture analysis of the tumors on the T2-weighted MR images. We found that 5 out of 25 wavelet-transform-energy-based features had a high potential to discriminate between tumors treated with orlistat and those without treatment. AUC = 1 was found for the features WavEnLL_s1 and WavEnHL_s4, which means 100% sensitivity and 100% specificity for differentiation between the control and treatment groups. These results, however, must be interpreted with caution due to the small sample size in our experiment. Wavelet transform analyzes the frequency content of an image at different scales. The wavelet decomposition of an image is carried out by applying a pair of quadrature mirror filters, a high-pass filter, and a low-pass filter. After signal decomposition, a set of spatially oriented frequency channels is available, which is used to describe local image variability. The energies within the frequency channels are then used as features. High-pass filtering in both directions captures diagonal details; high-pass filtering followed by low-pass filtering captures vertical edges; low-pass filtering followed by high-pass filtering captures horizontal edges; and low-pass filtering in both directions captures the lowest frequencies, at different scales [33]. Wavelet transform features express the homogeneity/heterogeneity of the internal structures of the examined tissue (mostly tumors), whereby edge structures are recognized by low-pass filtering and homogeneity by high-pass filtering.

Consistently, other studies also found a strong correlation between the T2-weighted MRI wavelet transform features and the number of Ki67 cells/field for the detection of early immunotherapeutic response following dendritic cell vaccine treatment in a mouse model of pancreatic ductal adenocarcinoma [34]. Li et al. reported that wavelet transform texture features demonstrated the potential to assist patient stratification for determining the suitability of liver resection vs. transcatheter arterial chemoembolization in patients with hepatocellular carcinoma [35]. Convolutional neural networks (CNNs) in combination with wavelet transform parameters have also been used to distinguish gliomas from other brain pathologies [36]. Tumor heterogeneity assessed by the visual or texture analysis of images should not be confounded with tumor heterogeneity based on differing gene expression, as we did not examine this relationship [37,38,39,40]. Histological inhomogeneity based on the criteria cell density, necrosis, new vessel formation, and fibrosis, however, represents the morphological substrate of the wavelet transform energy parameters.

### Limitations

Firstly, the environment of a heterotopically implanted xenograft differs fundamentally from that of one implanted orthotopically into the brain, mainly because the method does not reflect the complex interactions of glioma cells with the surrounding brain tissue in the immune microenvironment. In addition, it does not reproduce the intracerebral infiltrative tumor growth behavior in glioblastomas. However, the observation period in such an experimental setting would have been significantly shorter due to anatomical restrictions Nonetheless, applying an orthotopic xenograft model would have given the study more strength and should be the next step in future studies. Secondly, future studies should apply advanced MR techniques with functional and metabolic imaging in addition to morphological imaging to better detect and understand the (patho)physiological processes. In addition, MR examinations should be performed more frequently and earlier to monitor these processes. Thirdly, the groups in this study were small, thus raising concerns about the power of the study. However, guided by ethical concerns against the use of animals, our study was designed to reduce the number of animals used to the absolutely necessary minimum to meet scientific purposes.

## 5. Conclusions

This in vivo study shows discrepancies between the histologic and metabolic results, on the one hand, and MRI analysis after treatment with orlistat, on the other. While proliferative rates and apoptosis rates based on laboratory data did not differ significantly between the treatment and control groups, the interior structure and the pattern of tumor growth assessed by MRI were distinctive. The results suggest that, after an initial inhibitory effect of orlistat on cell proliferation, alternative metabolic pathways via cholesterol synthesis may have paved the way for tumor regrowth. Western blotting clearly showed that FASN expression was significantly inhibited in the orlistat group. Further studies should investigate the effects of FASN inhibition on glioblastoma when combined with targeted therapies.

## Figures and Tables

**Table 1:** Selection of the pulse sequence parameters used in the experiments.

	T2-SPACE	T2-SPACE FS	T1-VIBE
Dimension	3D	3D	3D
Fat suppression	no	SPAIR	no
TR [ms]	2500	2500	16
TE [ms]	289	289	2.66
Flip angle	variable	variable	20.5
Echo train length	71	71	-
Matrix	192	192	224
FOV [mm]	50	50	54
FOV phase [%]	71.9	71.9	93.8
NEX	1	1	2
Bandwidth [Hz/Px]	145	145	280
Slice thickness [mm]	0.25	0.25	0.25
Slices in stack [nr]	120	120	128
Voxel size [mm]	0.25 × 0.3 × 0.3	0.25 × 0.3 × 0.3	0.2 × 0.2 × 0.3
Acquisition time [min]	8:47	8:47	8:44

Note. Abbreviations: SPACE = sampling perfection with application-optimized contrasts using different flip angle evolution; FS = fat suppression; VIBE = volumetric interpolated breath-hold examination; SPAIR = spectral attenuated inversion recovery; TR = repetition time; TE = echo time; FOV = field of view; NEX = number of excitations.

**Table 2:** Primary antibodies. The primary antibodies used are listed with the manufacturer (company name, location), the corresponding dilutions, and Cat#.

Antigen	Antibody	Species	Manufacturer	Dilution	Cat#
APAF1	Anti-APAF1 antibody	Rabbit	Abcam, Cambridge, UK	1:1000	ab32372
Caspase-3	Caspase-3 antibody	Rabbit	Cell Signaling Technology Inc., Danvers, MA, USA	1:1000	9332S
Caspase-8	Anti-Caspase-8 antibody	Rabbit	Abcam, Cambridge, UK	1:2000	ab184721
FASN	Purified Mouse Anti-Fatty Acid Synthase	Mouse	Becton Dickinson, Franklin Lakes, NJ, USA	1:1000	610963
GAPDH	Anti-GAPDH antibody	Mouse	Abcam, Cambridge, UK	1:5000	ab9484
IκB-alpha	Anti-IKB alpha (phospho S32 + S36)	Mouse	Abcam, Cambridge, UK	1:500	ab12135
NFκB p50	NFκB p50 Recombinant Polyclonal Antibody (24HCLC)	Rabbit	Thermo Fisher Scientific Inc., Langenselbold, Germany	1:100	710450
NFκB p105	NFκB p105 Recombinant Polyclonal Antibody	Rabbit	Thermo Fisher Scientific Inc., Langenselbold, Germany	1:1000	23576-1-AP
PARP1	Anti-Poly (ADP-Ribose) Polymerase	Rabbit	Roche Diagnostic GmbH, Mannheim, Germany	1:2000	11835238001

**Table 3:** Secondary antibodies. The secondary conjugated antibodies used are listed with the manufacturer (company name, location), the corresponding dilutions, and Cat#.

Antibody	Manufacturer	Dilution	Cat#
Donkey anti-rabbit IgG-HRP	Santa Cruz Biotechnology, Inc., Paso Robles, CA, USA	1:5000	sc-2313
Goat anti-mouse IgG-HRP	Santa Cruz Biotechnology, Inc., Paso Robles, CA, USA	1:5000	sc-2024

**Table 4:** Overview of the results of visual qualitative MR analysis.

**Timepoint of Randomization**								
Mouse No.	Enhancement quality	Proportion of enhancement	Proportion of necrosis	SI rim T1 nativ	Thickness of contrast-enhancing rim	Pattern of tumor growth	Hemorrhage	Neovascular proliferation
A 1	mild	2/3	0	iso	thick	concentric	no	no
A 2	marked	3/3	0	iso	thick	concentric	no	yes
A 3	mild	3/3	0	iso	thick	concentric	yes	yes
A 4	mild	3/3	0	iso	thick	concentric	no	yes
A 5	mild	3/3	0	hypo	thick	concentric	no	no
A 6	marked	3/3	0	iso	thick	concentric	no	no
A 7	n|a	n|a	0	iso	n|a	concentric	no	no
B 1	marked	3/3	0	iso	thin	concentric	no	no
B 2	n|a	n|a	0	iso	n|a	concentric	no	no
B 3	mild	3/3	0	iso	thick	concentric	no	no
B 4	mild	3/3	0	iso	thick	concentric	no	no
B 5	marked	1/3	0	iso	thick	concentric	no	no
B 6	marked	2/3	0	iso	thick	concentric	no	no
**Final MR Examination**								
Mouse No.	Enhancement quality	Proportion of enhancement	Proportion of necrosis	SI rim T1 nativ	Thickness of contrast- enhancing rim	Pattern of tumor growth	Hemorrhage	Neovascular proliferation
A 1	mild	3/3	0	iso	thick	concentric	no	yes
A 2	mild	2/3	1/3	iso	thick	concentric	no	yes
A 3	marked	2/3	1/3	iso	thick	concentric	yes	yes
A 4	marked	2/3	1/3	iso	thick	concentric	no	yes
A 5	mild	3/3	0	iso	thick	concentric	no	no
A 6	marked	2/3	0	iso	thick	concentric	no	no
A 7	n|a	n|a	0	iso	n|a	concentric	no	no
B 1	marked	2/3	0	iso	thick	eccentric	no	no
B 2	n|a	n|a	0	iso	n|a	eccentric	no	no
B 3	marked	3/3	0	iso	thick	eccentric	no	no
B 4	marked	3/3	0	iso	thick	eccentric	no	yes
B 5	marked	1/3	0	iso	thick	eccentric	no	no
B 6	marked	2/3	0	iso	thick	eccentric	no	no

**Note.** First column: A = control group; B = treatment group. Abbreviations: n|a = not applicable; iso = isointense; hypo = hypointense. Only the pattern of tumor growth differs between the groups at the final MR examination.

**Table 5:** Overview of the results of histological and immunocytochemical analysis.

Mouse No.	Necrosis	Vessel Proliferation	Apoptosis	Ki67 [%]	GFAP	MAP2	p53 [%]
A 1	no	yes	scattered	10	n|a	n|a	10
A 2	yes	yes	scattered	30	+	+	20
A 3	yes	yes	scattered	40	−	−	10
A 4	yes	yes	scattered	30	−	+	40
A 5	no	no	no	n|e	+	+	−
A 6	no	no	no	20	−	−	5
A 7	no	no	no	20	−	−	10
B 1	no	no	no	90	+++	+++	40
B 2	no	no	no	70	+++	+++	40
B 3	no	no	scattered	20	−	+	10
B 4	no	yes	scattered	20	+	−	15
B 5	no	no	no	20	−	+	5
B 6	no	no	no	20	−	−	5

**Note.** First column: A = control group; B = treatment group. Abbreviations: n|a = not applicable; − = no immunoreactivity; + = weak immunoreactivity in less than 10% of cells; +++ = strong immunoreactivity in more than 80% of cells; n|e = not possible to evaluate.

## Data Availability

The data presented in this study are available upon request from the corresponding author.

## Supplementary Materials

The following supporting information can be downloaded at: https://www.mdpi.com/article/10.3390/cancers16081591/s1. Figure S1: Compilation of the eight Western blots of one or more mice-the number of the mouse is indicated in the upper row in the left picture.

## Abbreviations

CE	contrast-enhanced
FASN	fatty acid synthase
FFA	free fatty acids
GBM	glioblastoma multiforme
Gd DTPA	gadolinium diethylenetriaminepentaacetic acid
GFAP	glial fibrillary acidic protein
MR	magnetic resonance
MRI	magnetic resonance imaging
ROI	region of interest
SI	signal intensity
T	Tesla
TSE	turbo spin echo
T1w	T1-weighted
T2w	T2-weighted
