# Supplementary material for: Effects of Tetrahydrolipstatin on Glioblastoma in Mice: MRI-Based Morphologic and Texture Analysis Correlated with Histopathology and Immunochemistry Findings—A Pilot Study
Source: Cancers (Basel). 2024 Apr 21;16(8):1591. doi: 10.3390/cancers16081591 (PMC11048907; doi:10.3390/cancers16081591)
Supplement: Supplementary file 1 [file cancers-16-01591-s001.zip › cancers-2954948-supplementary.pdf]

Supplement:

The present study was a subproject of an orlistat treatment study, a total of 34 mice were examined: At the beginning of the project, as a pilot study, we performed MRI twice on nine consecutive mice, the first at the time of randomization and the second immediately before the animals were euthanized for histological and immunochemical work up. The xenografts were established into both flanks of the animals (treatment group 5 mice, unilateral: bilateral tumor growth 4:1; control group 4 mice, unilateral: bilateral tumor growth 1:3). These results form the basis of the present study. No MRI examinations were carried out on the following 25 mice in the further subproject.

All mice used in our pilot study are clearly labeled in the Western blots.

A

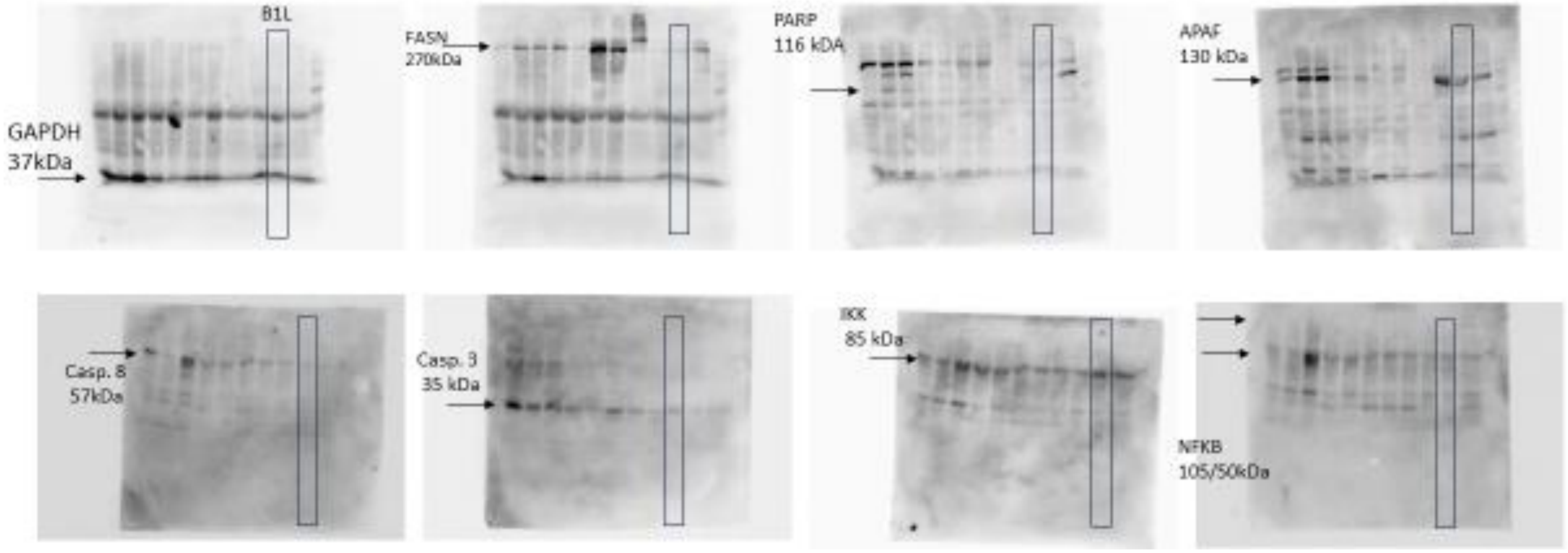

B

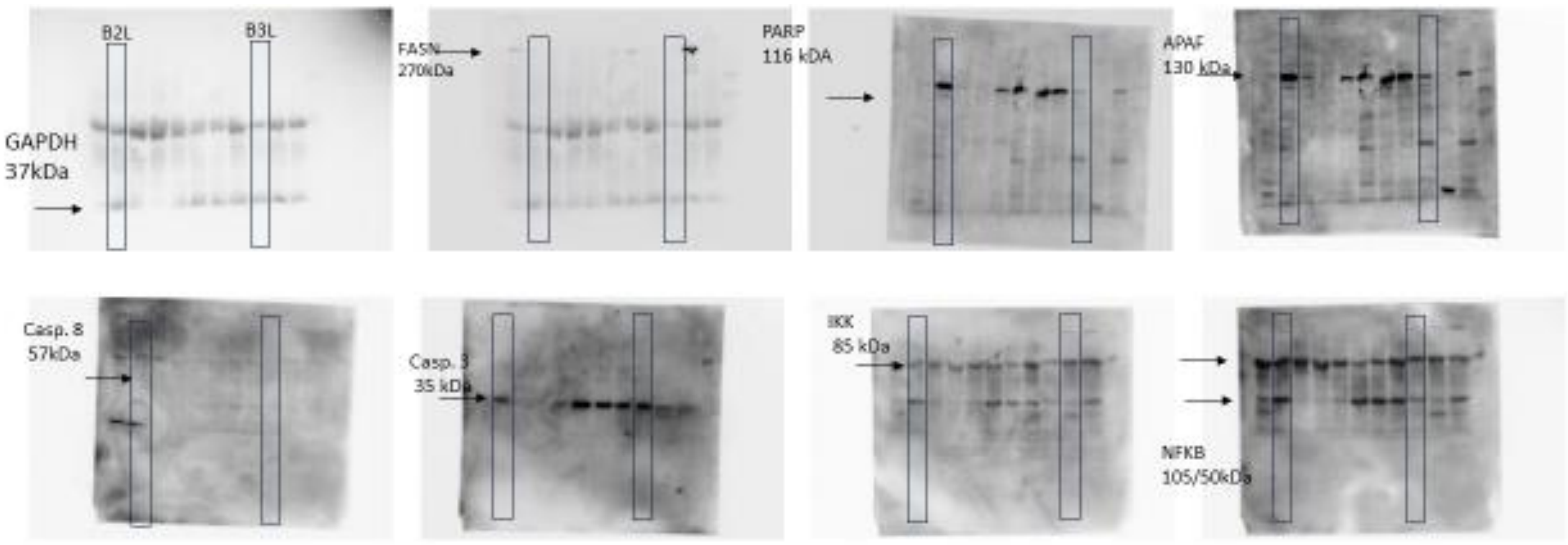

B2L
B3L
GAPDH
37kDa
FASN
270kDa
PARP
116 kDA
APAF
130 kDa
Casp. 8
57kDa
Casp. 3
35 kDa
IKK
85 kDa
NFKB
105/50kDa

C

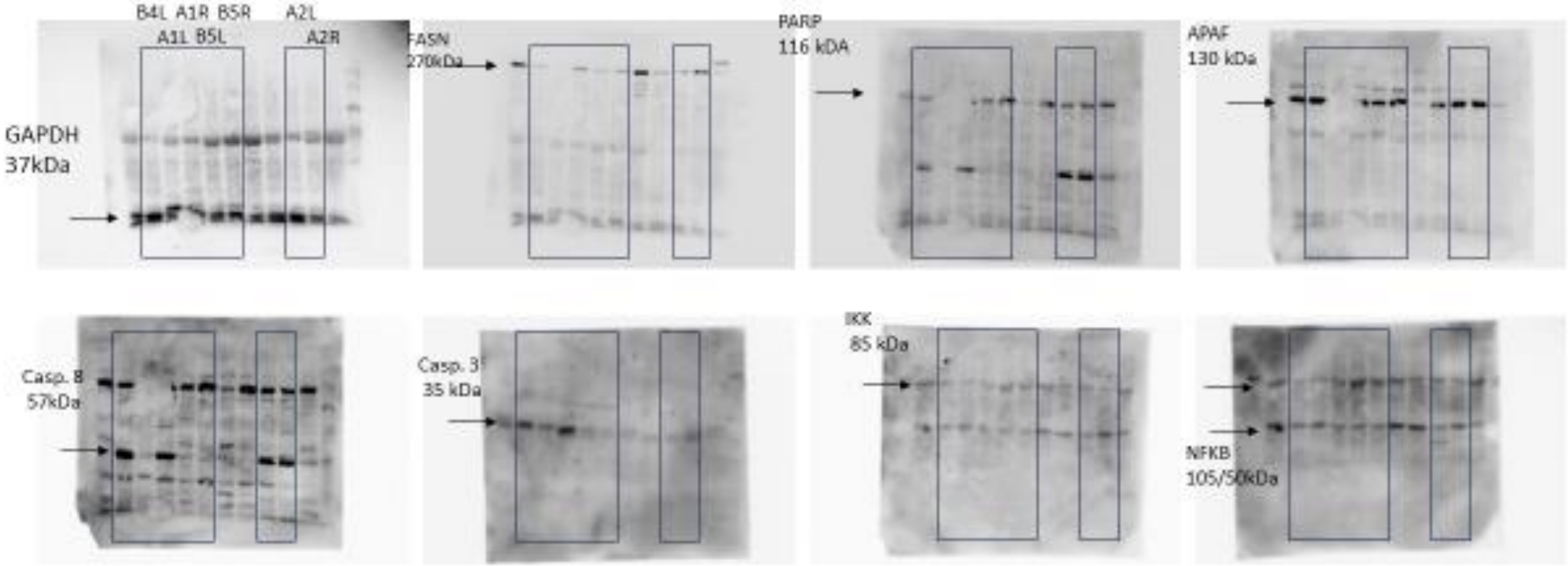
B4L A1R B5R A2L
A1L B5L A2R
GAPDH
37kDa
FASN
270kDa
PARP
116 kDA
APAF
130 kDa
Casp. 8
57kDa
Casp. 3
35 kDa
IKK
85 kDa
NFKB
105/50kDa

D

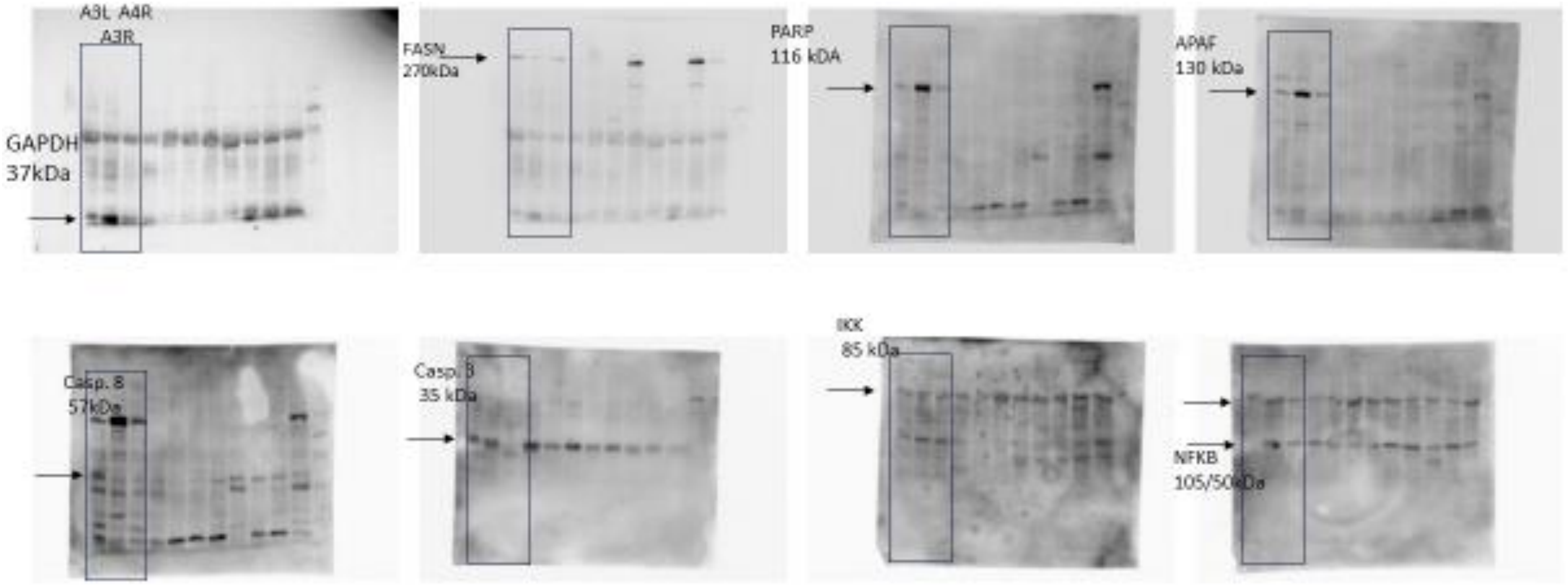

Supplementary Figure S1: (**A-D**) Compilation of the eight Western blots of one or more mice - the number of the mouse is indicated in the upper row in the left picture. **Note.** A = control group; B = treatment group; R = right; L = left (the xenografts were established into both flanks of the animals). Abbreviations: GAPDH = Glyceraldehyde-3-phosphate dehydrogenase; FASN = fatty acid synthase; PARP-1 = poly-adenosine diphosphate ribose polymerase-1; APAF1 = apoptotic protease activating factor-1; Casp. 8 = apoptosis-regulating proteins caspase-8; Casp.3 = apoptosis-regulating proteins caspase-3; IKK = IkappaB kinase; NF-κB = nuclear factor kappa-light-chain-enhancer of activated B-cells.
